# Microbiology-Based Instruction during Prenatal Dental Visits Improves Perinatal Oral Health Literacy

**DOI:** 10.3390/ijerph19052633

**Published:** 2022-02-24

**Authors:** Joshua J. Thomson, Erin E. Relich, John R. Girdwood, Divesh Byrappagari

**Affiliations:** 1 Division of Integrated Biomedical Sciences, University of Detroit Mercy School of Dentistry, Detroit, MI 48062, USA; 2 Division of Dental Hygiene, University of Detroit Mercy School of Dentistry, Detroit, MI 48062, USA; relicherin@gmail.com; 3 Division of Student Affairs, University of Michigan-Flint, Flint, MI 48502, USA; johngird@umich.edu; 4 Dental Public Health and Outreach, University of Detroit Mercy School of Dentistry, Detroit, MI 48062, USA; byrappdi@udmercy.edu

**Keywords:** maternal oral health, *Streptococcus mutans*, oral health literacy, dental, pregnancy, oral hygiene education

## Abstract

To improve oral hygiene education, we evaluated the perception and potential impact of microbiology-focused oral hygiene instructions (OHI) given to pregnant patients. Dental hygienists provided this supplemental education and administered Saliva-Check Mutans (SCM) tests to pregnant patients (*n* = 188) in Obstetrics and Gynecology (OB/GYN) settings. Patients reported their self-perceived understanding of the relationship between oral bacteria and dental disease and returned postdelivery to receive a second SCM test and follow-up questionnaire (*n* = 47). Prior to the hygienist instruction, 84% of participants understood that bacteria caused tooth decay, while only 36% understood they could transfer these bacteria to their children. After instruction, patient understanding increased to 97% and 95%, respectively. Participants attributed these increases to the hygienist’s explanation and SCM test. In postdelivery participants, >80% reported adherence to routine oral hygiene practices, and a significant decrease in patients with high-risk levels of salivary *Streptococcus mutans* was determined by SCM test (*p* = 0.0253). Participants agreed that the SCM test (89%) and microbiology explanation (95%) should be provided to every pregnant patient. Evaluation of patient perception of this intervention highlights how focused instruction on the infectious nature of dental disease can increase perinatal oral health literacy. Microbiology-focused education should be given to pregnant patients to reduce oral health disparities.

## 1. Introduction

Elimination of oral health disparities is crucial in the fight to improve oral and overall health. Improvement in patient education about dental disease and oral hygiene must be included in the approach towards eliminating oral health disparities [1]. Despite the growing body of evidence supporting the safety and importance of providing dental care to pregnant patients, dental care is accessed less frequently during pregnancy, mostly by low-income, Medicaid-eligible pregnant people with limited access to dental care during pregnancy [2]. Perinatal oral health literacy is important to guide family oral health [3]. For example, children of mothers with untreated caries or high levels of tooth loss are more likely to have higher levels of caries experience [4]. With overall less access to dental care during pregnancy, there is less opportunity for patient education at this critical time, so interventions are needed. 

During dental visits, oral health education generally covers the importance of routine at-home toothbrushing and interdental cleaning, regular dental visits, reduction of sugar consumption, and consideration of certain lifestyle changes [5]. Yet, these topics do not include specific education regarding the microbial processes underpinning the pathogenesis of these diseases. Patients lacking knowledge about the role of bacteria in dental diseases may not fully realize the necessity of these routine oral hygiene practices. Thus, patient education highlighting that oral hygiene practices are intended to reduce the progression of an infectious disease process may provide better motivation for a patient to adhere to the described oral health care recommendations.

Caries remains one of the most common chronic diseases of childhood and can have serious consequences in child development [1,6]. Initiation of caries onset is highly correlated with the presence of the acid-producing bacterium, *Streptococcus mutans* [7]. Generally, *S. mutans* begins to colonize hard surfaces in the oral cavity as teeth begin to erupt [7,8,9], and the initial acquisition of *S. mutans* by a child is most frequently from the saliva of the mother or primary caretaker [10,11,12]. Directed education to pregnant patients on these bacteria-driven processes may be especially valuable because salivary *S. mutans* levels in mothers are correlated with increased levels of colonization of tooth surfaces by *S. mutans* in their child, which is subsequently correlated with increased risk of caries incidence during childhood [8,13]. Additionally, transfer of *S. mutans* to an infant can be delayed through the reduction in maternal *S. mutans* levels, and delayed colonization in the child is associated with decreased risk of caries development [14,15,16]. Enhanced understanding of the bacterial component of this disease and of the bacterial transmission from parent to child may incentivize pregnant patients to follow routine dental hygiene practices to reduce their levels of *S. mutans*.

Therefore, the objective of this study was to assess patient perception of hygiene education during pregnancy customized towards understanding the role of bacteria in dental disease and the importance of routine dental hygiene practices to maintain low levels of colonization on teeth. In this study, dental hygienists placed in Obstetrics and Gynecology (OB/GYN) settings provided pregnant patients with focused explanations of the role of *S. mutans* in dental disease along with the risk of transmission of these bacteria from primary caretaker to a child. To further augment the explanation, a chairside test (Saliva-Check Mutans [SCM]) was administered to quickly determine the level of salivary *S. mutans*. This educational intervention was provided with the intent that parents would understand the infectious disease process and thus appreciate the importance of oral hygiene, not only for their own oral health but also for the oral health of their children.

## 2. Materials and Methods

### 2.1. Ethical Considerations

This study was reviewed and approved by the Institutional Review Board of the University of Detroit Mercy (Protocol #1718-24) and the Michigan Department of Health and Human Services (IRB Log #: 201712-06-EA). Informed consent was received from all subjects in this study. 

### 2.2. Placement of Dental Hygienists

Dental hygienists were provided dedicated space in OB/GYN settings at six different Federally Qualified Health Centers (FQHCs) in Michigan as part of the Michigan Initiative for Maternal and Infant Oral Health (MIMIOH) project. This project was designed to provide prenatal dental care to pregnant people and facilitate their transition to a permanent dental healthcare home. Four of the six health centers participated in the present study: Grace Health (Battle Creek, MI, USA), Intercare Community Health Network (Benton Harbor, MI, USA), Ingham Community Health Centers, Cedar Campus-Women’s Health (Lansing, MI, USA), and Great Lakes Bay Health Centers-Women’s Care (Bay City, MI, USA).

### 2.3. Subject Enrollment

In the present study, a total of 188 pregnant individuals (at any point during their pregnancy) were recruited. Participants were patients of record at the included OB/GYN settings or new patients at any point during their pregnancy. There were no exclusion criteria. Participants recruited at these health centers received supportive health insurance through Medicaid and thus were considered at high caries risk due to the high correlation between economically disadvantaged status and caries prevalence [17,18,19]. 

### 2.4. Experimental Overview

**Figure 1:**
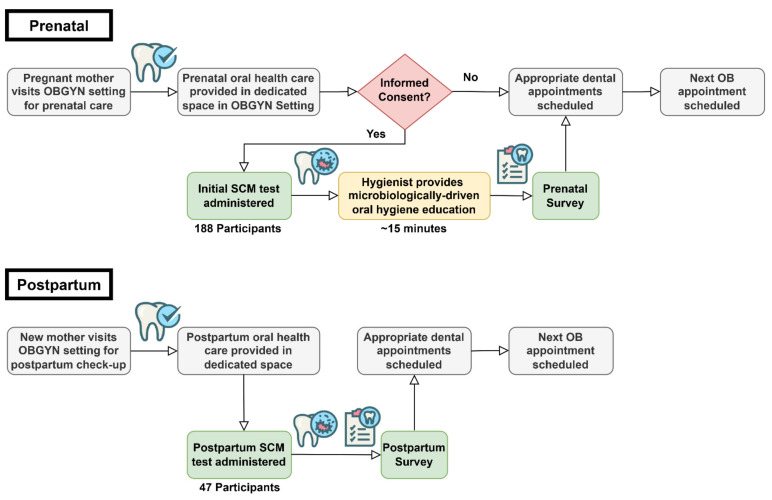
Intervention sequence to provide oral health instruction using SCM test and microbiology-based education during the prenatal dental screening at an OB/GYN setting and during the follow-up screening postdelivery (postpartum). Dental hygienists were given dedicated space in OB/GYN clinics to provide dental screenings to pregnant patients and find them a dental home. In this space, they received informed consent and proceeded with the administration of the SCM test and microbiology-based oral health education, followed by a prenatal questionnaire. After delivery, a postpartum screen was administered, and a follow-up SCM test and questionnaire were given. Abbreviations: OBGYN = Obstetrics and Gynecology; SCM = Saliva-Check Mutans.

A flow chart for the educational intervention is shown in Figure 1. Briefly, pregnant patients were enrolled and given a prenatal education session with a hygienist, took a chairside, Saliva-Check Mutans (SCM) test (GC-America, approximate cost per test USD 15.00), and completed a prenatal questionnaire. Patients returning after giving birth took another *S. mutans* test and completed a postpartum questionnaire. 

### 2.5. Prenatal Intervention

The hygienist conducted an initial dental screening while the pregnant patient waited to see the doctor or immediately after their appointment (Figure 1). During this first visit, after informed consent, the patient provided a stimulated saliva sample after chewing on a wax pellet from the kit, and the hygienist processed the SCM test according to the manufacturer’s instructions. 

During the test’s 15-min run time, the hygienist provided guided education about bacteria and dental disease using a laminated handout (Figure S1). The hygienist’s explanation covered four microbiological topics related to oral disease. These topics were: (1) The relationship between cavity-causing bacteria in plaque and cavities (tooth decay); (2) Routine oral hygiene practices such as brushing, flossing, and regular dental cleanings are performed to remove cavity-causing bacteria from the surface of teeth; (3) Primary caretakers are usually responsible for the early transmission of cavity-causing bacteria to their children; and (4) Routine oral hygiene practices such as brushing, flossing, and regular dental prophylaxis in the primary caretaker can reduce transmission to their children and positively impact their oral health.

Using the handout (Figure S1), the hygienist described how certain bacteria in plaque, principally *S. mutans*, produce acid from dietary sugars, which leads to cavities. Then, they explained why the reduction of dietary sugars decreases the amount of bacteria-generated acids. Next, disclosing solution photographs were used to display bacterial biofilm accumulation (plaque) on teeth. Hygienists discussed how routine oral hygiene practices are essential to remove plaque which would decrease the number of bacteria and acid production after consuming sugar. Finally, patients were educated on the transmission of *S. mutans* from primary caretaker to a child and how delayed transmission can positively impact the child’s oral health. At the conclusion, the hygienist summarized that the best way to delay or prevent the transmission of bacteria to their children was by lowering their own levels through strict adherence to routine oral hygiene practices.

SCM test results were interpreted according to manufacturer’s instructions: a positive result indicated ≥5.0 × 10^5^ CFU/mL salivary *S. mutans*, while a negative result indicated <5.0 × 10^5^ CFU/mL salivary *S. mutans* [20,21]. If the test was positive, the hygienist explained that improving oral hygiene practices could result in lower levels of *S. mutans*. If negative, the hygienist would explain the importance of continuing proper oral hygiene practices to maintain low *S. mutans* levels. Following this interaction, the patients completed a 26-question prenatal survey (Initial Survey). 

### 2.6. Postpartum Intervention

At the first postpartum visit to the OB/GYN, the hygienist met with the returning patient and performed a second SCM test. The patients completed a follow-up 18-question questionnaire (Postpartum survey) designed to evaluate their perception of the usage of the SCM kit and hygienist intervention, as well as their reported adherence to oral hygiene practices proposed in the initial survey. 

### 2.7. Questionnaire Administration

The prenatal and postpartum questionnaires (Figures S2 and S3) were hosted by Qualtrics^XM^. The questionnaire was administered electronically on a tablet in the presence of the hygienist to provide reading assistance.

### 2.8. Calibration of Dental Hygienists

The hygienists were briefed on the aim and scope of the research project and were provided with calibration materials, including the oral health handout, a patient interaction flowchart, and a calibration checklist (Figure S4). Additionally, a mock patient interaction video was created by the authors to illustrate the intended flow of the educational intervention. 

### 2.9. Statistical Analysis

The change in the proportion of positive to negative tests for the pregnant people with paired tests was determined with Statistical Package for the Social Sciences (SPSS) Statistics 26 (IBM; Armonk, NY, USA) using the McNemar test with and without Yates’ correction for continuity (*p* < 0.05). 

## 3. Results

**Figure 2:**
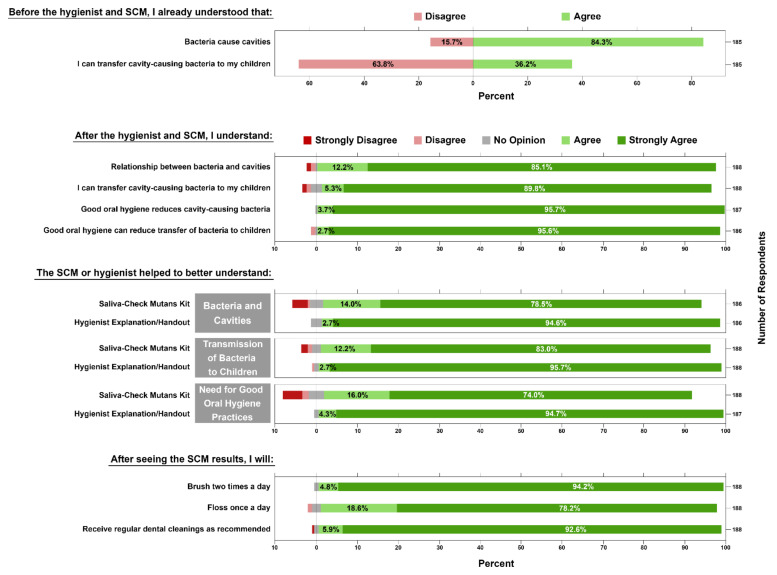
Compiled results of the prenatal questionnaire. Patients were asked to agree or disagree with their perceived knowledge of the microbial role in causing cavities. Following questions in the survey used a 5-point Likert scale (1: Strongly Agree to 5: Strongly Disagree) to gauge patients’ self-reported level of understanding of various microbiology-based oral health statements. Using the same scale, patients were also asked to report agreement with whether the Saliva-Check Mutans (SCM) test or hygienist explanation/handout helped them to understand the previous educational points. Finally, patients were asked if they planned to maintain routine oral hygiene practices after seeing their SCM test result. Figure created using Inkscape.

Following the evidence-based educational session, the hygienist distributed a questionnaire to assess the patient’s level of understanding about the role of bacteria in dental disease, as well as to ascertain their feelings about the components of this educational intervention approach. A summary of the results of the prenatal (initial) survey is presented (Figure 2). This initial survey was given to 188 patients, 126 (67.0%) between 0 and 20 weeks pregnant and 62 (33.0%) between 21 and 40 weeks pregnant. The survey contained questions to determine whether they brushed (82%), flossed (14%), used mouth rinse (24%) during the current day, or had been on antibiotics in the last month (16%). 

The survey was constructed to assess the patients’ previous knowledge about two main topics explored during the educational session using dichotomous “agree/disagree” questions. Of the 188 participants, 156 (84.3%) agreed that they already understood that bacteria caused cavities and tooth decay before the educational intervention, while only 67 (36.2%) agreed that they had a prior understanding that they could transfer cavity-causing bacteria to their children (Figure 2). However, these positive responses may not reflect true levels of prior understanding since the survey was given after the educational intervention in the presence of the hygienist. These circumstances could have unintentionally led a patient to put an affirmative answer on the survey. 

The survey also consisted of a series of 5-point Likert scale questions (Strongly Agree = 1, Agree = 2, No Opinion = 3, Disagree = 4, Strongly Disagree = 5) to ascertain the patients’ understanding of all four educational topics following the SCM test and hygiene education session. There was an increase from 84.3% of participants with prior understanding up to 97.3% (Agree + Strongly Agree) current understanding of the relationship between bacteria and cavities, and from 36.2% to 95.2% in the understanding of their role in transferring cavity-causing bacteria to their children. Additionally, participants near-unanimously agreed (Agree + Strongly Agree) that they now understood how good oral hygiene practices could reduce cavity-causing bacteria in their mouth (99.5%) and reduce bacterial transmission to their children (98.4%). 

Participants were asked separate questions about whether the hygienist explanation/handout and the SCM test helped them to understand these educational points using the same 5-point Likert scale. Participants responded with an overall agreement (Agree + Strongly Agree) to both education points, with a slightly increased influence from the explanation/handout (Figure 2). The frequency of “Strongly Agree” was increased for the hygienist explanation/handout compared with the SCM test, respectively, for the influence on the participant’s understanding of the relationship between bacteria and caries (94.6%, 78.5%), of the transfer of cavity-causing bacteria (95.7%, 83.0%), and of the need for adherence to good oral hygiene practices (94.7%, 73.9%). Lastly, the survey measured the participants’ level of agreement to brush twice a day (94.1% Strongly Agree), floss at least once a day (78.2% Strongly Agree), and receive regular dental prophylaxis as recommended (92.6% Strongly Agree). 

**Figure 3:**
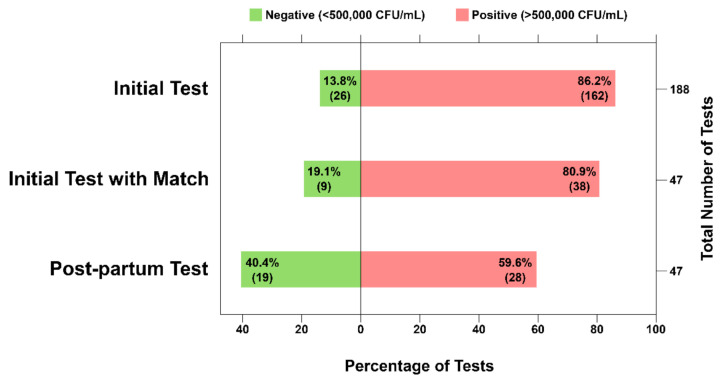
Saliva-Check Mutans (SCM) results from the prenatal (Initial) test and Postpartum SCM test. An SCM test was administered during the hygienist dental screening of pregnant patients during a prenatal visit (*n* = 188, “Initial Test”) and screening after delivery (*n* = 47, “Postpartum Test”) to qualitatively measure the *S. mutans* salivary concentration. “Initial test with Match” indicates prenatal/initial SCM test results of participants that had a paired SCM test from returning postdelivery. Bars represent the percentage of positive and negative results in each group. Figure created using Inkscape. Abbreviations: CFU = colony-forming units.

In the initial/prenatal test group, there were 162/188 (86.2%) positive SCM tests (Figure 3, Initial Test). Of the 188 participants that received initial tests, 47 (25.0%) returned postpartum and received a second test. In the returning group, 30 (63.8%) were between 0–5 weeks postdelivery. Of this returning group, 38 (80.9%) received a positive initial SCM test (Figure 3, Initial Test with Match). For the returning postpartum test, the overall percentage of positive tests was reduced to 28 (59.6%). Of the 38 participants with positive initial SCM tests, 23 (60.5%) were also positive in the postpartum test, while 15 (39.5%) had reduced salivary *S. mutans* indicated by a negative test result (Table 1). This difference was statistically significant using the McNemar test with (*p* = 0.0253) and without (*p* = 0.0442) Yates’ correction for continuity. It is important to note that this significant difference cannot be attributed to the intervention by microbial education alone since the participant return rate was 25%, and possible additional dental interventions were not identified in this study. However, these data provide evidence of positive change in oral hygiene along with positive feedback from the participants regarding their understanding of disease processes.

**Figure 4:**
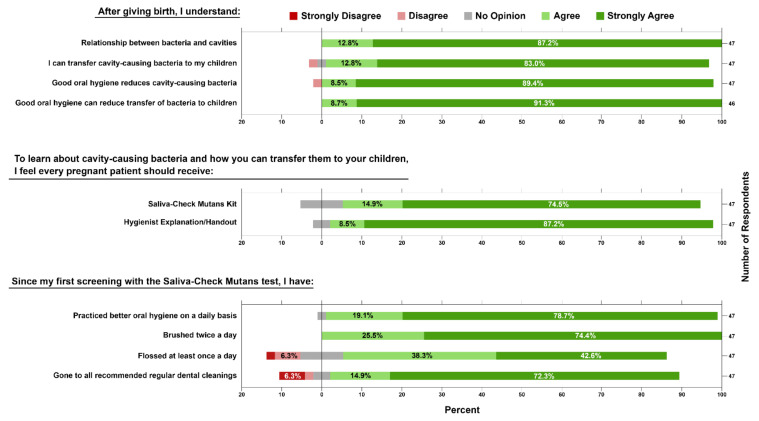
Compiled results of the postpartum questionnaire. Patients have answered a questionnaire that utilized a 5-point Likert scale (1: Strongly Agree to 5: Strongly Disagree). The first set of questions addressed patients’ level of agreement with affirmative statements that they understood the four microbiology-based oral health statements from the prenatal survey. They were then asked to use the Likert scale to answer if they believed the Saliva-Check Mutans (SCM) or specific hygiene explanation/handout should be given to every pregnant patient. Finally, patients were asked to self-report their agreement with statements about their adherence to routine oral hygiene practices since the prenatal screening. Figure created using Inkscape.

In the postpartum questionnaire, patients were asked about their understanding of the same educational topics as in the initial survey using a 5-point Likert scale. Patients maintained strong agreement that they still understood each of the four educational points (Figure 4). Additionally, they were asked if they felt every pregnant patient should be given the SCM test (89.4%, Agree + Strongly Agree) and if the hygienist should describe the role of cavity-causing bacteria and risk of transmission from caretaker to child to every pregnant patient (95.7%, Agree + Strongly Agree). Then, they were asked to self-report their level of agreement with statements about their adherence to oral hygiene practices since receiving their first SCM test, and 46/47 (97.9%) agreed that they had practiced better oral hygiene daily. Specifically, 100% agreed that they brushed twice a day, 80.9% had an agreement with flossing at least once a day, and 87.2% had an agreement with going to all recommended dental cleaning/prophylaxis appointments.

## 4. Discussion

*S. mutans* salivary levels, while highly correlated with the initiation of caries and caries-active patients, lack accuracy in predicting future caries for the whole population [22]. Nevertheless, *S. mutans* bacterial load assessment can be a useful tool to illustrate bacterial presence on teeth as part of an overall caries risk assessment and an indication of antimicrobial treatment success [22,23,24]. The semiquantitative Saliva-Check Mutans (SCM) test has been shown to reliably and accurately detect salivary *S. mutans* in 30 min [20,21]. Since the measurement of *S. mutans* bacterial load is not sufficient for caries risk assessment, we attempted to highlight an alternative utility of this bacterial chairside test by combining its use with specific microbiology-focused oral health education. As shown in our study, this educational intervention was perceived by patients as helpful and increased their self-reported knowledge of the role of bacteria in caries (Figure 2 and Figure 4). A vast majority of participants agreed to maintain directed oral hygiene practices such as brushing, flossing, and attending dental appointments as advised (Figure 2). Upon a follow-up postpartum visit, a significant portion of returning participants had reduced salivary *S. mutans* levels, possibly due to increased adherence to routine oral hygiene practices (Table 1, Figure 3). These are indications that perinatal oral health literacy was increased as a result of this educational intervention.

Transmission of *S. mutans* from mother to child was an important educational component in the hygienist’s explanation since transmission to the child is most frequent from the primary caretaker [10,11,12], and early age infection with *S. mutans* is a significant risk factor for future dental caries [25,26,27]. Additional studies have shown a significant correlation of increased salivary *S. mutans* levels in mothers with detection of *S. mutans* in the saliva of infants with 6–8 primary incisors [14]. The frequency of colonization in the infant was approximately nine times greater when the mother harbored salivary counts more than 10^5^ CFU/mL compared with mothers with counts less than 10^3^ CFU/mL [16]. Therefore, it is encouraging that we observed a decrease in the proportion of positive (>10^5^ CFU/mL) SCM tests in the postpartum period after the educational intervention by the hygienist (Table 1, Figure 3). 

The need for perinatal oral health education is highlighted in a multicenter study of 262 pregnant patients, where the authors illustrate poor patient knowledge and behavior regarding perinatal oral health [28]. Educational interventions designed to improve the oral health of the mother, reduce maternal bacterial levels and promote behavior modeling that can subsequently improve the children’s oral health outcomes [29]. Nevertheless, this education mostly consists of instruction on oral hygiene practices and feeding/dietary habits. In a clinical trial in Hong Kong [30], researchers provided family-centered behavioral and educational counseling during the first-time pregnancy with follow-ups throughout the first two years of the child’s life. In this trial, test group toddlers had significantly better oral hygiene practices, feeding habits, and overall oral health status, including lower *S. mutans* levels [30]. In our study, education was specifically designed to describe the microbiological basis of dental disease and to demonstrate existing bacterial levels using a chairside diagnostic kit (Figure 1). Participants reported strong agreement that both handout/hygienist explanation and SCM test helped in their understanding of the four educational points about bacteria and dental disease, corroborated by the reduction in salivary *S. mutans* levels shown by the SCM tests (Figure 2 and Figure 3). The positive responses from our study participants to the microbiology-focused education and SCM test demonstration/discussion illustrate benefits on their own, but more so if included in the sessions described in the Hong Kong trial [30].

Lastly, our study further validates the role of dental hygienists outside the traditional setting of clinical dental practice. Our results show that dental hygienists working in an OB/GYN alternative practice setting are an asset in providing educational oral health instructions which benefit both mother and child. Collaborative multidisciplinary medical group practices should consider recruiting dental hygienists to assist their patients in reducing risk for disease transmission and improving oral health.

## 5. Limitations of the Study

This study was not a clinical trial, and there was no control group to determine the level of education on disease processes with standard oral hygiene education. The questionnaire provided at each visit relied on self-reporting, which is inherently skewed. Response to the questionnaire was also conducted in the presence of the hygienist. Since some questions pertained to the explanation provided by the hygienist, participants may have felt inclined to answer affirmatively to those questions. This study was also limited by a 25% return rate for postpartum visits. This study did not assess whether participants had exposure to any other oral health education before the follow-up visit postdelivery, which could affect these findings.

## 6. Conclusions

In this study, patients reported a new and continued understanding of the process of dental disease and the importance of oral hygiene practices to control these disease processes. Both the microbiological explanation by the hygienist and the use of the SCM test kit was reported to aid in participant understanding of dental disease processes, with almost all reporting that this type of evidence-based education should be provided to all pregnant patients. This study highlights how focused instruction on the infectious disease process can increase perinatal oral health literacy. More similar large-scale studies are needed to determine if early educational interventions combined with routine SCM testing in alternative practice settings (FQHCs or OB/GYN offices) will benefit mothers and their unborn children.

## Figures and Tables

**Table 1:** Results of paired prenatal (initial) and postpartum Saliva-Check Mutans (SCM) tests of participants that returned postpartum.

		Postpartum SCM Result		
		Positive	Negative	Total
Initial SCM Result	Positive	23	15 *	38
	Negative	5	4	9
	Total	28	19	47

* Statistically significant increase in Negative SCM test results in participants that had initial positive results with (*p* = 0.0253) and without (*p* = 0.0442) correction for continuity using the McNemar test. Positive: ≥5.0 × 10^5^ CFU/mL salivary *S. mutans*. Negative: <5.0 × 10^5^ CFU/mL salivary *S. mutans.*

## Data Availability

The raw data are available from the corresponding author upon request.

## Supplementary Materials

The following supporting information can be downloaded at: https://www.mdpi.com/article/10.3390/ijerph19052633/s1, Figure S1: Microbiology of dental disease handout; Figure S2: Prenatal Questionnaire, Figure S3: Postpartum Questionnaire, Figure S4: Hygienist Checklist.
