# Supplementary material for: Microbiology-Based Instruction during Prenatal Dental Visits Improves Perinatal Oral Health Literacy
Source: Int J Environ Res Public Health. 2022 Feb 24;19(5):2633. doi: 10.3390/ijerph19052633 (PMC8910304; doi:10.3390/ijerph19052633)
Supplement: Supplementary file 1 [file ijerph-19-02633-s001.zip › Figure S1 - Handout_Takehome - Images Removed.pdf]

Healthy teeth versus teeth with plaque

DENTAL BIOFILM
(PLAQUE)

Healthy Tooth

Insert Picture of healthy tooth with no visible plaque

Brushing, flossing & use of mouth rinse 2 times daily

Tooth with Dental Biofilm (Plaque)

Insert Picture of tooth with visible plaque biofilm and gingival inflammation

Lack/infrequency of daily brushing, flossing and mouth rinse

Streptococcus mutans is a bacterium that initiates tooth decay

Saliva-Check Mutans measures amount of *S. mutans* in your mouth

Bacteria in plaque + sugar makes acid that eats away at teeth (tooth decay)

Explanation on oral hygiene & best practices

Oral hygiene is the practice of keeping one's mouth clean and free of disease and other problems by **regular brushing and cleaning between the teeth**.

It is important that oral hygiene be carried out on a regular basis to enable prevention of dental disease. **Oral hygiene helps remove cavity-causing bacteria from your mouth**.

The most common types of dental disease are tooth decay and gum diseases, including gingivitis, and periodontitis.

Regular brushing consists of **brushing twice a day** after breakfast and before going to bed. **Brushing removes plaque (cavity-causing bacteria) from surface of teeth**.

Cleaning between the teeth is called interdental cleaning **and is as important as tooth brushing**. This is because a toothbrush cannot reach between the teeth and therefore, only cleans 50% of the surfaces. There are many tools to clean between the teeth, including **floss and interdental brushes**. It is up to each individual to choose which tool he or she prefers to use.

Antibacterial mouth rinses can also be used to keep breath fresh and **kill the bacteria** in the mouth that causes oral diseases.

Insert image of teeth prior to application of disclosing solution

Insert image of teeth after application of disclosing solution showing areas of plaque accumulation

BEFORE & AFTER disclosing solution/colored dye applied

- Plaque (bacterial biofilm) is not always easy to detect as it usually blends in with the tooth
- By looking under a microscope or by using colored disclosing solution/dyes, plaque is quite noticeable!
- Plaque (bacterial biofilm) returns rapidly (within 8-12 hours), so **frequent removal by brushing, flossing and mouth rinse is key!**

How can cavity causing bacteria be transferred from a mother to her child?

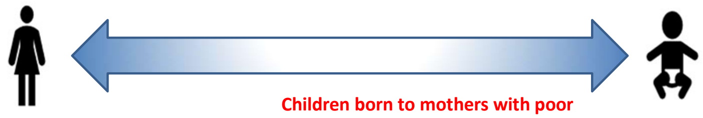

Children born to mothers with poor oral health have higher risk of early childhood tooth decay

How can mom reduce transmission of bacteria to child?

1.________________

2.________________

3.________________

4.________________

5.________________

Remove bacteria in mouth = Better oral health of the mother = better oral health of the child

Insert images of toothbrushes, floss, and mouthrinse

Supplemental Figure S1
