# Supplementary material for: Microbiology-Based Instruction during Prenatal Dental Visits Improves Perinatal Oral Health Literacy
Source: Int J Environ Res Public Health. 2022 Feb 24;19(5):2633. doi: 10.3390/ijerph19052633 (PMC8910304; doi:10.3390/ijerph19052633)
Supplement: Supplementary file 1 [file ijerph-19-02633-s001.zip › Figure S2 - Prenatal Survey from Qualtrics.pdf]

Figure S2. Prenatal Questionnaire

Default Question Block

What is your research number?

How many weeks pregnant are you?

What was the result of the Saliva-Check Mutans test?

- Positive (strong band on "T")
- Partial Positive (weak band on "T")
- Negative (No band on "T")

Have you brushed your teeth today?

- Yes
- No

Have you flossed your teeth today?

- Yes
- No

Have you used mouthrinse today (e.g. Listerine)?

- Yes
- No

Have you used antibiotics in the last month?

- Yes
- Maybe
- No

Indicate your level of agreement or disagreement with the following statements regarding your understanding of tooth decay before this visit:

I already understood that bacteria caused cavities and tooth decay before this visit.

- Strongly agree
- Somewhat agree
- Neither agree nor disagree
- Somewhat disagree
- Strongly disagree

I already understood that cavity-causing bacteria could be transferred to my children.

- Strongly agree
- Somewhat agree
- Neither agree nor disagree
- Somewhat disagree
- Strongly disagree

Indicate your level of agreement or disagreement with the following statements regarding your understanding after meeting with the hygienist:

I understand the relationship between bacteria and cavities (tooth decay).

- Strongly agree
- Somewhat agree
- Neither agree nor disagree
- Somewhat disagree
- Strongly disagree

I understand that I can transfer cavity-causing bacteria to my children.

- Strongly agree
- Somewhat agree
- Neither agree nor disagree
- Somewhat disagree
- Strongly disagree

I understand that I can reduce cavity-causing bacteria in my mouth with good oral hygiene practices like brushing, flossing, and regular dental cleanings.

- Strongly agree
- Somewhat agree
- Neither agree nor disagree
- Somewhat disagree
- Strongly disagree

I understand that a good way to reduce transmission of cavity-causing bacteria to my children is to have good oral hygiene practices (brushing, flossing, etc.).

- Strongly agree
- Somewhat agree
- Neither agree nor disagree
- Somewhat disagree
- Strongly disagree

The **Saliva-Check Mutans test** helped me to understand the relationship between bacteria and tooth decay.

- Strongly agree
- Somewhat agree
- Neither agree nor disagree
- Somewhat disagree
- Strongly disagree

The **Saliva-Check Mutans test** helped me to understand that cavity-causing bacteria can be transferred to my children.

- Strongly agree
- Somewhat agree
- Neither agree nor disagree
- Somewhat disagree
- Strongly disagree

The **Saliva-Check Mutans test** was necessary for me to better understand a need for good oral hygiene practices.

- Strongly agree
- Somewhat agree
- Neither agree nor disagree
- Somewhat disagree
- Strongly disagree

The **explanation/handout given by the hygienist** helped me to understand the relationship between bacteria and cavities (tooth decay).

- Strongly agree
- Somewhat agree
- Neither agree nor disagree

- Somewhat disagree
- Strongly disagree

The **explanation/handout given by the hygienist** helped me to better understand that cavity causing bacteria can be transferred to my children.

- Strongly agree
- Somewhat agree
- Neither agree nor disagree
- Somewhat disagree
- Strongly disagree

The **explanation/handout given by the hygienist** helped me to better understand the need for good oral hygiene practices.

- Strongly agree
- Somewhat agree
- Neither agree nor disagree
- Somewhat disagree
- Strongly disagree

Which was **MOST** helpful for your understanding of cavity-causing bacteria and good dental health?

- Saliva-Check Mutans Test
- Hygienist Explanation/Handout
- Both the test and hygienist were most helpful together
- Neither the test nor the hygienist were helpful

Now that I have seen the result of my Saliva-Check Mutans test:

I will make sure to brush 2 times a day.

- Strongly agree
- Somewhat agree
- Neither agree nor disagree
- Somewhat disagree
- Strongly disagree

I will floss at least once a day.

- Strongly agree
- Somewhat agree
- Neither agree nor disagree
- Somewhat disagree
- Strongly disagree

I will make sure to receive regular dental cleanings as recommended.

- Strongly agree
- Somewhat agree
- Neither agree nor disagree
- Somewhat disagree
- Strongly disagree

Powered by Qualtrics
