# Supplementary material for: Microbiology-Based Instruction during Prenatal Dental Visits Improves Perinatal Oral Health Literacy
Source: Int J Environ Res Public Health. 2022 Feb 24;19(5):2633. doi: 10.3390/ijerph19052633 (PMC8910304; doi:10.3390/ijerph19052633)
Supplement: Supplementary file 1 [file ijerph-19-02633-s001.zip › Figure S3 - Postpartum Survey from Qualtrics.pdf]

Figure S3. Postpartum Questionnaire

Default Question Block

What is your research number?

How many weeks pregnant are you?

What is the result of Saliva-Check Mutans test?

- Positive (strong band on "T")
- Partial Positive (weak band on "T")
- Negative (No band on "T")

Have you brushed your teeth today?

- Yes
- No

Have you used mouthrinse today (e.g. Listerine)?

- Yes
- No

Have you used antibiotics in the last month?

- Yes
- Maybe
- No

Indicate your level of agreement or disagreement with the following statements:

I understand the relationship between bacteria and cavities (tooth decay)

- Strongly agree
- Somewhat agree
- Neither agree nor disagree
- Somewhat disagree
- Strongly disagree

I understand that I can transfer cavity-causing bacteria to my children

- Strongly agree
- Somewhat agree
- Neither agree nor disagree
- Somewhat disagree
- Strongly disagree

I understand that I can reduce cavity-causing bacteria in my mouth with good oral hygiene practices like brushing, flossing, and dental cleanings.

- Strongly agree
- Somewhat agree
- Neither agree nor disagree
- Somewhat disagree
- Strongly disagree

I understand that a good way to reduce transmission of cavity-causing bacteria to my children is to have good oral hygiene practices (brushing, flossing, etc.).

- Strongly agree
- Somewhat agree
- Neither agree nor disagree
- Somewhat disagree
- Strongly disagree

I feel that the Saliva-Check Mutans kit should be given to every pregnant patient to learn about cavity-causing bacteria and the risk of transmission to their children.

- Strongly agree
- Somewhat agree
- Neither agree nor disagree
- Somewhat disagree
- Strongly disagree

I feel that the hygienist should describe the role of cavity-causing bacteria to every pregnant patient to help learn about tooth decay and the risk of transmission of bacteria to children.

- Strongly agree
- Somewhat agree
- Neither agree nor disagree
- Somewhat disagree
- Strongly disagree

Since my first screening, with the Saliva-Check Mutans test, I have practiced better oral hygiene on a daily basis.

- Strongly agree
- Somewhat agree

- Neither agree nor disagree
- Somewhat disagree
- Strongly disagree

Since my first Saliva-Check Mutans test during my initial screening:

I have brushed twice a day

- Strongly agree
- Somewhat agree
- Neither agree nor disagree
- Somewhat disagree
- Strongly disagree

I have flossed at least once a day.

- Strongly agree
- Somewhat agree
- Neither agree nor disagree
- Somewhat disagree
- Strongly disagree

I have gone to all recommended regular dental cleaning appointments.

- Strongly agree
- Somewhat agree
- Neither agree nor disagree
- Somewhat disagree
- Strongly disagree

Since your first Saliva-Check Mutans test during your initial screening:

Powered by Qualtrics
