# Supplementary material for: Microbiology-Based Instruction during Prenatal Dental Visits Improves Perinatal Oral Health Literacy
Source: Int J Environ Res Public Health. 2022 Feb 24;19(5):2633. doi: 10.3390/ijerph19052633 (PMC8910304; doi:10.3390/ijerph19052633)
Supplement: Supplementary file 1 [file ijerph-19-02633-s001.zip › Figure S4 - Hygienist Checklist.pdf]

Research Project Calibration Checklist

CHECKLIST FOR SUCCESSFUL IMPLEMENTATION

TASK	DONE	INITIALS
Patient informed consent (discuss, sign & collect)<br>***Patient to receive a copy of the informed consent**.		
Collect saliva and perform Saliva-Check Mutans (SCM) test.<br>Mention test measures the amount of a cavity-causing bacterium called *Streptococcus mutans*		
While test is processing (approximately 15 min.), discuss plaque, bacteria and oral diseases (the bacteria/plaque on teeth eat sugars which makes acid that can eat away at the teeth/enamel). Discuss how we should remove the plaque and bacteria. Also, discuss how the bacteria can be transmitted to a child. Write down the patient responses to these scenarios on the take home handout for the patient. (See PowerPoint Slides (39-42 from calibration session for specific content)		
<u>Check results of SCM test and inform patient</u>:<br>**positive (+) OR negative (-)** ***Should always be a band at "C"**		
**(+) If test is positive (band at "T"), discuss how to bring bacteria levels down and why it is important from a disease transmission standpoint**		
**(-) If test is negative (No band at "T"), discuss what the patient must be doing correctly (daily brushing, flossing, use of mouth rinse) and stress that they keep these behaviors up for optimal oral health and reduction of bacterial/disease transmission to a child**		
**On the Pregnancy Saliva-Check Pre-Survey**, the supervising dental hygienist needs to provide the research number, **the week of pregnancy, and the result of the SCM test (positive or negative).**		
Turn the computer towards the patient and let them click their responses to the remaining questions on the survey. You can assist with reading if needed.		
At the end of the session, mention the second SCM test at the postpartum visit (after the baby is born)		
**<u>BE SURE YOU KEEP THE SIGNED COPY OF THE INFORMED CONSENT TO BE GIVEN TO THE RESEARCHERS.</u> THE PATIENT TAKES HOME A COPY OF THE INFORMED CONSENT PAPERWORK AND A COPY OF THE ORAL HYGIENE HANDOUT/WORKSHEET! Thank you ☺**		

Supplemental Figure S2
